# Climate change, culture and health: Indigenous resilience, a study from Turkana County, Kenya

**DOI:** 10.4102/jamba.v16i1.1647

**Published:** 2024-08-13

**Authors:** Christian Muragijimana, Theoneste Ntakirutimana, Sohaib Khan

**Affiliations:** 1 Department of Public Health, Faculty of Health Sciences, University of Eastern Finland, Kuopio, Finland; 2 Department of Environmental Health Sciences, School of Public Health, College of Medicine and Health Sciences, University of Rwanda, Kigali, Rwanda

**Keywords:** droughts, indigenous knowledge, health, disaster risk reduction, resilience, Turkana, arid and semi-arid lands

## Abstract

Climate change and recurring droughts-induced effects on health are becoming an increasingly main global, cultural and public health burden. The heaviest health burden leans on the fragile socio-economic systems among the remote agro-pastoral communities, living in the arid and semi-arid lands (ASALs). Previous studies underlined the indispensability of indigenous knowledge (IK) for resilience-driven disaster risk reduction (DRR) strategies. However, more attention has been drawn towards the necessity of IK in weather forecasts, with less emphasis on its indispensability to alleviate health burden associated with climate change and droughts. We explored the contextual application of IK-based adaptation and related complementarity aspects for culturally relevant and sustainable DRR strategies for the nomadic agro-pastoral communities in Lopur, Turkana, Kenya. Relying on a descriptive qualitative study in phenomenological approach, purposive sampling and focus group discussions with key community influencers, a thematic analysis was conducted for an in-depth understanding and interpretation of data patterns. The contextualised insights revealed the growing vulnerability as a result of the disconnect between modern interventions, IK and the newly adopted environmental degrading coping tactics. Policy-wise, the findings portrayed the necessity for cultural integration and incorporation of indigenous knowledge-based strategies and systems for reinforced information dissemination, accessibility and acceptability for droughts preparedness and response.

**Contribution:**

This study underlined the existing room for scientific exploration of the already existing indigenous knowledge-based solutions for food and water insecurity, towards improved resilience for the vulnerable communities experiencing inequitable climate change calamities in the ASALs.

## Introduction

Drought is a slow-onset disaster characterised by a dry prolonged period in the natural climate cycle (WHO 2024). Based on its increasing frequency, severity and pervasiveness, droughts are becoming one of the major global, social and public health threats. Droughts, as the leading cause of water scarcity, impact 40% of the world’s population, with ruinous effects on mass migration in which 700 million people are at risk of displacement by 2030 (WHO 2024). Health disparities, through the lens of limited resources, fragile and unsustainable measures to implement and reinforce adaptation strategies compared to developed countries, make the developing world, and the arid and semi-arid lands (ASALs) in particular, more vulnerable to the aforementioned calamities (Parkes 2006; WHO 2012).

Various studies (Iloka 2016; Masinde 2015; Pelser 2001; Rukema & Umubyeyi 2019; Theodory 2020) have underpinned the necessity and associated constraints for integrating indigenous knowledge (IK) as the foundational and vital element for sustainable local-based policies to mitigate the pernicious health and livelihoods effects linked with recurring droughts. However, the existing evidence principally reflects on the complementarity and reliability of indigenous knowledge forecasts (IKFs) and scientific knowledge (SK) in weather and climate forecasts.

**FIGURE 1:**
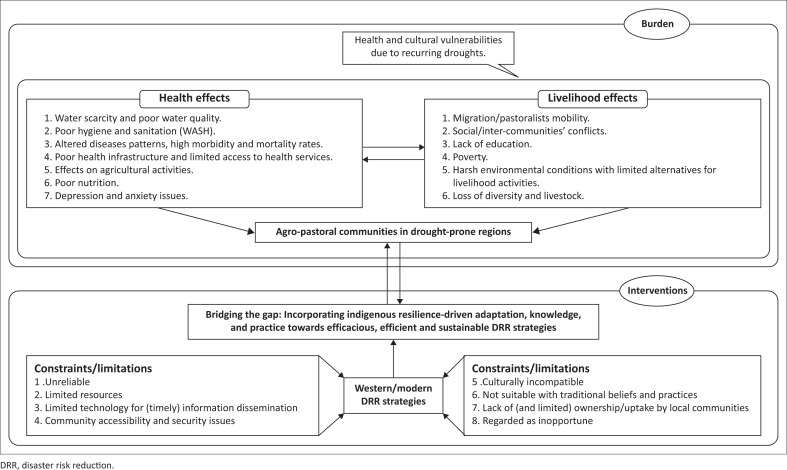
Conceptual framework.

Stemming from the conceptual framework (Figure 1) and the reviewed literature (County Government of Turkana 2014, 2016; Fatehpanah et al. 2020; Grey, Masunungure & Manyani 2020; Iloka 2016; Kagunyu, Wandibba & Wanjohi 2016; Masinde 2015; Mutu 2017; Opiyo et al. 2015; Rukema & Umubyeyi 2019; Theodory 2020; Waila et al. 2018; WHO 2012, 2024; Zvobgo et al. 2022), this paper explored the IK-driven adaptation strategies as well as practices of this knowledge. Further, this study casts light on IK complementarity aspects for adoptable, culturally relevant and sustainable ‘modern’ disaster risk reduction (DRR) strategies based on shared contextual insights from Lopur, an agro-pastoral community in early transition from nomadism to a settled and semi-settled lifestyle in Turkana County, Kenya.

The main aim of this study was to cast light on the necessity of IK and practices towards more sustainable and culturally friendly DRR strategies. The intent was to contribute to research, policy and adjusted practices to adapt to drought-induced effects on health and livelihoods, precisely in the ASALs’ remote drought-prone agro-pastoral communities. Specifically, this study explored:

- effects of recurring droughts on health and livelihoods;
- indigenous knowledge and practice to alleviate the aforementioned burden on culture and public health; and
- acceptability, suitability and adjustments necessity of modern DRR strategies through the lens of IK.

## Research methods and design

We applied qualitative descriptive study in phenomenological approach to comprehend the unique lived local experiences and used purposive sampling method based on the key intent of this study, which was to explicitly describe the contextual conditions pertinent to our main research objective. Further, this was considered to be a reliable strategy based on its key characteristics that mainly focus on the ‘meaning’ of the experience in question and the ‘analogy’ behind it (or the ‘what is it like to have such an experience’) (Cypress 2018).

Kenya, as one among the low- and middle-income countries (LMICs) in the eastern coast of the African continent, is one of the developing countries that is severely affected by droughts. Listed among countries with the per capita water availability of 1000 m^3^ annually (below the 1700 m^3^ threshold), Kenya belongs in the category of water-scarce countries (Mulwa, Li & Fangninou 2021). Eighty per cent of the Kenyan total land is classified as ASALs, which makes it one of the countries that are highly susceptible to droughts, and an approximated 70% of all livestock and 30% of the total Kenyan population live in these drought-prone areas (Uhe et al. 2018).

Prone to reoccurrence of droughts and water scarcity (Opiyo et al. 2015), it can take up to 5 h to reach the water source at the temperature of around 90 °Fahrenheit in Turkana (Waila et al. 2018). Besides water scarcity, the Turkana communities are susceptible to various health effects including altered disease patterns associated with food and water borne-diseases, poor nutrition and sanitation, compromised developmental projects, loss of diversity and community displacement (County Government of Turkana 2014).

In contrast to transhumance, the traditional nomadic life of Turkana communities requires them to follow the irregular weather patterns looking for water and fresh pasture which have been linked to disease outbreaks and cattle raiding or conflicts between communities (Huho, Ngaira & Ogindo 2011; Mureithi & Opiyo 2010; Opiyo et al. 2015). Leading a water scarce and predominantly nomadic life, with little to no humanitarian assistance, the Turkana communities experience droughts disproportionately compared to other regions (Mutu 2017; Ouma, Obando & Koech 2012; Waila et al. 2018).

This study took place in Turkana South, Katilu Ward, within the Lopur agro-pastoral community. Through the consultation and guidance from Lopur community leaders and other local-based governmental entities, the recruitment of study participants reflected the local customs, norms and culture, which allowed open-ended responses from the participants and helped to acquire a better understanding portraying the on-ground realities ingrained in the lived experiences of the Lopur community members. This study intended to include half of both male and female participants. However, there was limited availability of female participants during the discussions. Nevertheless, the aforementioned criterion remained flexible (as it was anticipated) in the preparation stage as a component that would be highly dictated by the local norms, customs and beliefs. This study applied purposive sampling as a means to reflect on the key intent of this paper, in which the goal was a breadth of understanding of the main objective of the study by ensuring that participants with a perceived extensive expertise were included (Etikan 2017; Sharma 2017).

Through the help and guidance by the recruited research team members, a translator and a mobiliser, born and raised in Turkana, with similar cultural background as the participants, the study recruited 28 participants in total, that is 7 participants per each focus group discussion (FGD) (Ahmed 2021). This study focused on four FGDs with key community influencers including political leaders, religious leaders, opinion leaders and community elders who were mainly involved in local decision making and had the required firsthand knowledge and were willing to share their experiences with regard to IK and related application to alleviate social, cultural and health burden associated with climate change.

In addition, the inclusion criteria for FGD with community elders was based on being a native resident with 50 years and above (as the group anticipated to have the most related generational expertise), ability to recall the memories and methods and willingness to participate (Fatehpanah et al. 2020; Speranza et al. 2009). Besides field observations, the study also referred to existing secondary sources of data, such as information from local water department and other relevant institutions. Furthermore, it is worth noting that the number of the required participants and related on-site data collection practicalities were evaluated and adjusted by piloting the interviews in a convenient setting to test the interview guide, address logistical needs, ensure richness and meaningfulness of the acquired insights from the study participants (McLafferty 2004).

**FIGURE 2:**
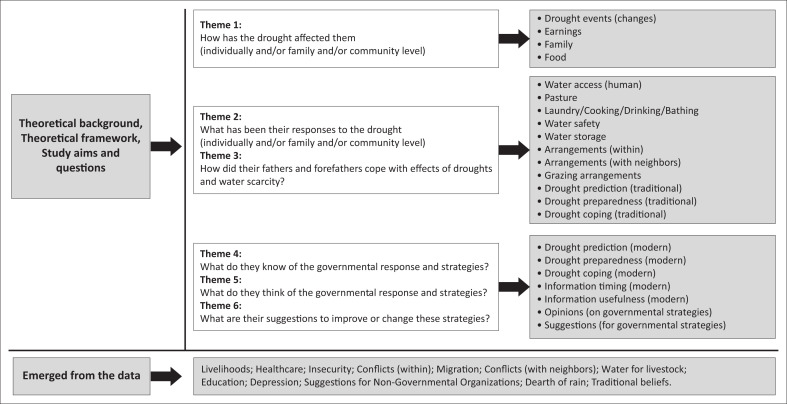
The emerged themes from the shared experiences.

The study prioritised the richness of data and flexibility during data collection. This was ensured through providing a comfortable, harmonious and synergetic environment to share and acquire a better understanding through open-ended questions that encouraged debate regarding beliefs, opinions, perspectives and experiences among the participants (Ahmed 2021; Mishra 2016). Primary data were collected through FGDs in Turkana (as the mother tongue of the participants), Swahili (as the Kenyan common local language) and English (as the most used Kenyan international language). The major six key domains (Figure 2) that were relied on included:

- how has the drought affected them individually and/or family and/or community level;
- what has been their responses to the drought (individually and/or family and/or community level);
- how did their fathers and forefathers cope with effects of droughts;
- what do they know of the governmental response and strategies;
- what do they think of the governmental response and strategies; and
- what are their suggestions to improve or change these strategies.

Prior to data collection, the team was informed that the first group discussion with community elders will be exclusively in Turkana language. As this was partially against the original plan, it introduced a complication regarding overcoming the barrier for the primary researcher (PR) who was in this instance an outsider. However, to avoid losing the meaning of original expressions through translation, the team addressed this issue by making sure that the conversation was not only between the translator and the participants but also between the PR and the translator.

The intent was to ensure real-time (on-field) clarification and confirmation of the experiences being shared based on not only the comfortability aspect of the participants but also the facial expression, body expression, the tone of the voice and other related field observations, to make sure the participants, the translator and the PR are all on the same page. The discussions were audio-recorded and kept by the PR. Each conducted interview was transcribed verbatim by the same PR, assisted by the translator and double-checked by the core researchers (Sartore et al. 2008). The research team ensured the related accuracy by making sure that the transcription was conducted at the highest detail level of the data (Terry et al. 2017). For a better understanding of the shared experiences, the team applied a hybrid technique built on ‘structured’ and ‘emerging’ coding techniques or deductive and inductive reasoning.

The deductive approach was applied to reinforce the relevancy and contribution of the open-coding to the produced themes and put an appropriate emphasis of the shared insights through the lens of the adopted research questions (Terry et al. 2017). Relying on generated and emerged themes from the transcripts, a thematic analysis (TA) was conducted (Swain 2018). In total, the team conducted two rounds of TA for around 2.5 weeks. The team ensured that the TA process is rather recursive and iterative than a linear analysis to accommodate for the possible necessity to move back and forth as necessary as per the followed TA analytical phases (Terry et al. 2017). The adopted TA analytical phases reflected the approach by Byrne, D (2022) and followed the proposed six TA steps:

- data engagement or familiarisation;
- coding or identification and lebelling of appropriate features in the data;
- creation of a robust thematic mapping;
- checking and reviewing if the created themes fit with the lebelled data and the logical process of the entire dataset. Further;
- the team proceeded with a brief but effective description and naming or difinition of themes; and
- the team reflected on the analytical narratives or compiled data extracts as part of the written report (Byrne 2022).

As part of the informed consent, the team requested and obtained permission from the participants (captured on records) after addressing key concerns related to anonymity in responding, voluntary participation and confidentiality in the related reports and field notes (while gathering, storing and handling data) throughout the study.

## Results

### Scientific knowledge-driven prediction and preparedness for droughts

As part of modern strategies, all the participants illustrated that they rely on technology, where the ‘weatherman’ informs them of the situation. Along the same vein, during our discussions with religious and opinion leaders, the participants stated that they rely on the ‘meteorological people’ and the ‘scientific things’ to know if rain is coming, or drought season is approaching:

**Quote 1:** ‘… the mterol … the meteorological people … like throwing balloons up there and bring information [---] that the rain will come, or the drought is… a severe drought is coming … so, we depend [---] we depend on scientific [*Sic*]… they say … these scientific things we depend on them… metrological … they are from the ministry of metrologies…’ (Religious Leader, Male, Respondent 4)

Concerning information timing and preparedness, during our discussion with community elders, a participant stated:

**Quote 2:** ‘… that message come in late time. Information comes late’ (Community Elder, Male, Respondent 1)

In the same vein, during our discussion with religious leaders, a participant stated:

**Quote 3:** ‘… if it [*the information*] could come earlier, someone could be able to prepare…’ (Religious Leader, Male, Respondent 6)

Underlining their views and beliefs regarding their forefathers’ indigenous knowledge-driven prediction of droughts, while discussing with opinion leaders, a participant stated:

**Quote 4:** ‘… and also, we still have our old elders… you know they said that a person who leaves his culture becomes a slave… there are those elders who still observe that intestines… intestines of goat…’ (Opinion Leader, Male, Respondent 2)

Demonstrating the indispensability of their culture in prediction of drought and rain seasons, the participants demonstrated that they still rely on their traditions (such as interpretation of intestines) regardless of and besides the existing modern strategies.

### Indigenous knowledge-driven prediction and preparedness for droughts

All the participants emphasised the use of goat intestines as a practice they inherited from their forefathers. They mentioned that this helps them to know not only if drought is coming, but also about the diseases, insecurity and if they will receive any external modern support or not. The participants demonstrated the process as they killed the goat, skinned, opened the stomach and interpreted the colour and status of the intestines. In our discussion with community elders (perceived by other groups as knowledge keepers), a participant stated:

**Quote 5:** ‘… [*in Turkana*] … translation … the way they open a book like this. They just open the intestine like this… so there are some, there are some spots, and there are some spots they see…’ (Community Elder, Male, Respondent 2)

As described, they open the intestines ‘like a book’ and read based on some spots. Elaborating more on the same, another participant (demonstrating to the group) added:

**Quote 6:** ‘… [*in Turkana*] … translation… so this is the intestine… if it drinks a water… water comes in… if the intestine is full of water, it turns red. And we say is full of water. If there is no water, it turns white, meaning there is no rain. [*S*]o, those elders they slaughtered an animal or a goat, so they read the, the small intestines. Matumbo [*small intestines*] they now read. There now they could see now there is a lot of drought…’ (Community Elder, Male, Respondent 1)

After reading and capturing the information, their elders make sure to pass it to the community through the community elders:

**Quote 7:** ‘So, they read and they tell the community … they will spread through these other old men, to each village …’ (Opinion Leader, Female, Respondent 6)

Elaborating more on this IK-based information sharing to prepare for droughts, the participants demonstrated that their forefathers used to receive the information from their interpreters such as diviners. In our discussion with community elders, a participant pointed out:

**Quote 8:** ‘… [*in Turkana*]… translation … he is saying… he is saying, so each and every one has to know… so that he or she can prepare as early as this for that drought. So these diviners were preparing them, in advance. So that they know what is going on’ (Community Elder, Male, Respondent 5)

Each and every one knew about what was happening and prepared based on this advance information received from the diviners. Along the same lines, during our discussion with political leaders, a participant added:

**Quote 9:** ‘…He moves around the Kraal, give announcement that prepare… prepare… and prepare adequately… the dry spell is coming’ (Political Leader, Male, Respondent 3)

So, the diviners would bring the information to the kraal (decentralised leadership of community segments) and inform them that drought is coming, and everyone got prepared adequately. Further, sharing more on the IK-based drought preparedness strategies, they included grazing patterns for the pasture and about their own food. They would cook meat until it becomes lean, dry it up and preserve it. Additionally, they also collected a lot of milk during the rainy season, boil and sun-dry and keep it in skin sacks to be used during droughts.

**PHOTO 1:**
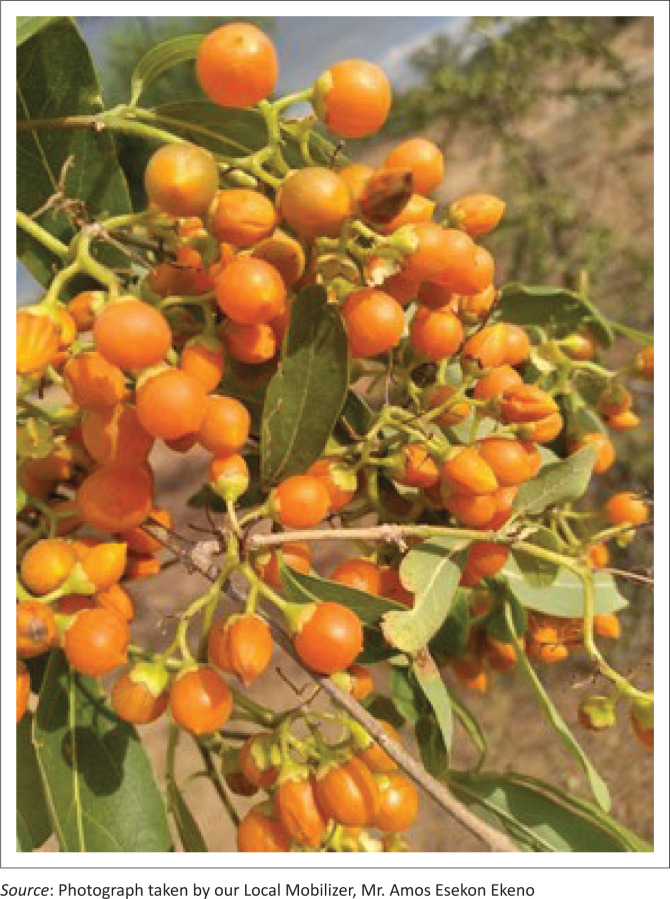
EDOME (*Cordia sinensis*).

**PHOTO 2:**
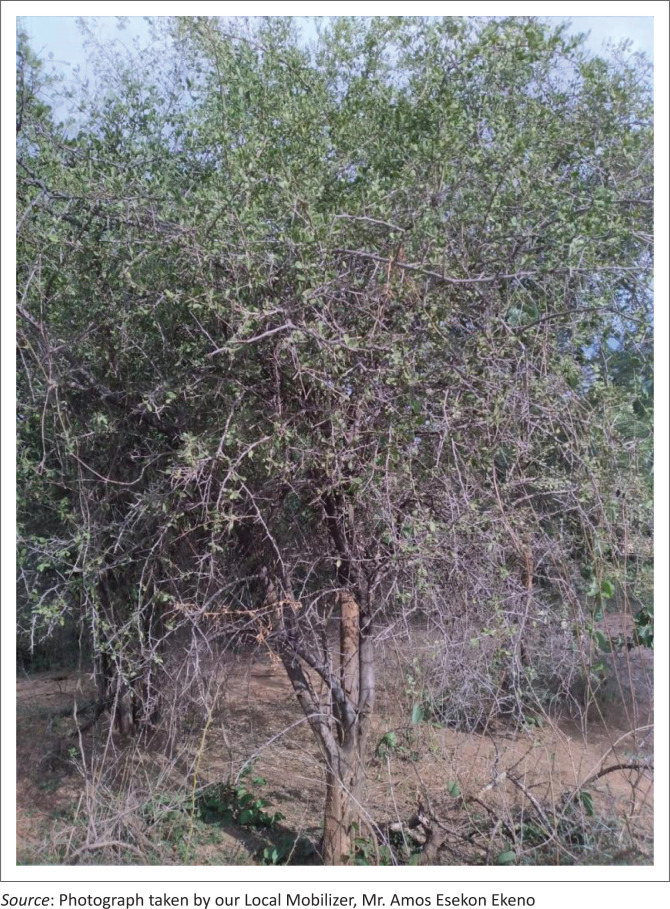
ELAMACH (*Balanites pedicullaris*) TREE.

**PHOTO 3:**
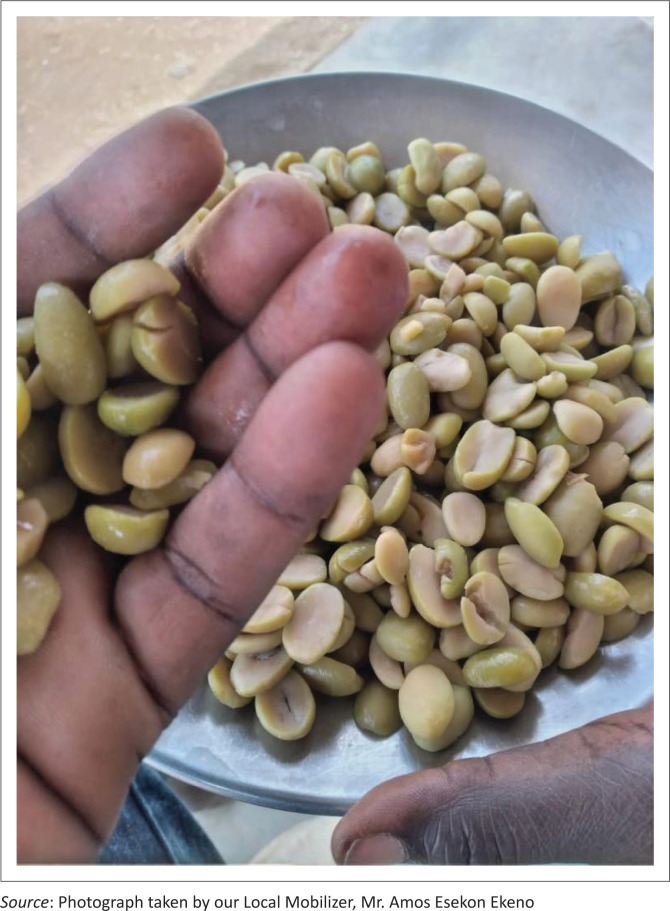
ELAMACH (*Balanites pedicullaris*).

The participants also mentioned that their fathers and forefathers used to collect, prepare and store wild fruits such as Edung (*Boscia coriacea*), Ng’akalalio (*Ziziphus mauritiana*), Edome (*Cordia sinensis*) (Photo 1) and Elamach (*Balanites pedicullaris*) (Photo 2 and Photo 3) to be consumed during drought (CGIAR 2023; Watkins 2010):

**Quote 10:** ‘…also before… our people, our elders, our forefathers they used to collect these local fruits… they collect EDOME [*Cordia sinensis*], ERUT [*Maerua decumbens*], ELAMACH [*Balanites pedicullaris*], wild fruits… there was those you boil… you boil like seven times, eight… then it becomes… the bitterness fade out. Now that one, when it is a lot, they dried it, and cook it, and when that bitterness is no longer there, they sundry it and keep in the … their sack… sack made in the skin of goat… they fill it … [*slapping hands*] …and store. That was for drought…’ (Religious Leader, Female, Respondent 2)

### Drought-induced burden on health

People mentioned the ‘decrease’ and ‘unpredictability’ of rainfall over time. The respondents pointed out that:

**Quote 11:** ‘… you know, maybe… maybe that one can be attribute to [---] the issue of climate change. … it is unpredictable, rain is unpredictable. You cannot predict like maybe in January or February you will get rain, you might even stay a whole year [---] without rain…’ (Religious Leader, Male, Respondent 5)

As demonstrated by the participant ‘maybe’ the aforementioned issue of small rains might be linked to climate change.

As far as health and well-being are concerned, referring to healthcare access, a participant, in our discussion with religious leaders, added:

**Quote 12:** ‘… if you want this help you get from someone for example from Lodwar, and you don’t have transport… to go there, or means of… any means… so, we can even live… with, that disease will make you to pass. Maybe you are diagnosed of a certain disease, and maybe the family lacks that power of taking you to the nearby hospitals that have such medicines…’ (Religious Leader, Male, Respondent 7)

Poverty, inadequate availability, affordability and accessibility of healthcare services are major concerns, where in some cases you have to travel all the way to Lodwar (the largest town in Turkana) to receive care. Besides issues related to accessing healthcare and poverty, people understood how droughts were predisposing them to diseases; during our discussion with opinion leaders, a participant stated:

**Quote 13:** ‘… [*in Swahili*] … it [*drought*] also brings diseases… there is a time… like right now during drought, you find somewhere there is water… it stays there for a long time, and because you are thirsty, you just drink that water, and you might get disease of Kipindupindu[*Sic*] Cholera, ehh… such kind of diseases…’ (Opinion Leader, Female, Respondent 4)

**PHOTO 4:**
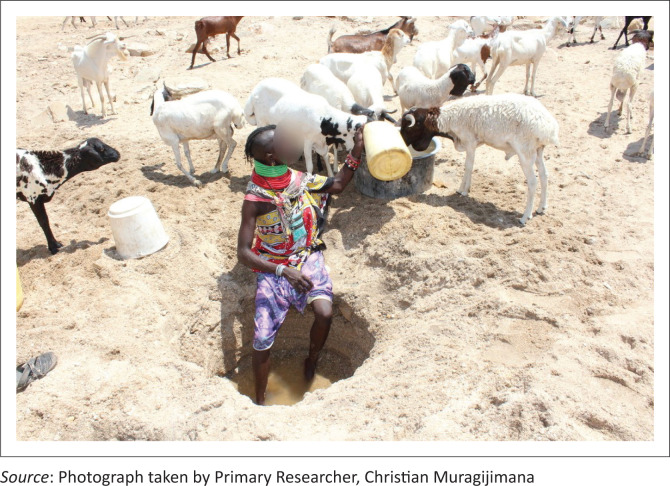
Traditional hand-dug well.

**PHOTO 5:**
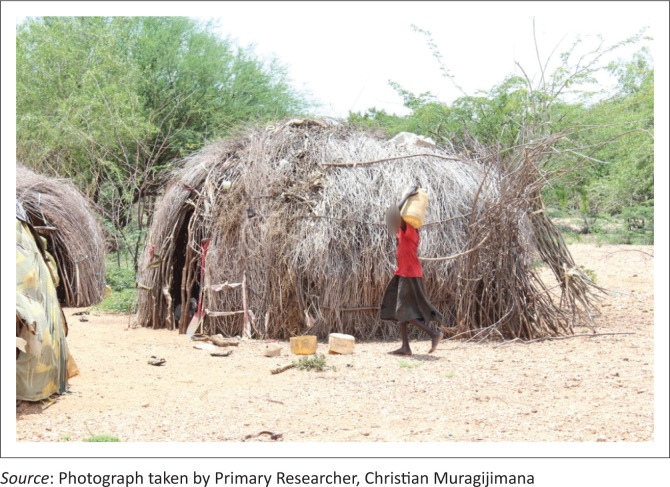
A Lopur young girl carrying water for household use.

As part of IK-driven practices to access water during drought, as shared by other groups, during our discussion with community elders, the participants described the process of accessing water and sharing it among themselves:

**Quote 14:** ‘…So, during the drought they will move to that area, so that they access water from there. They can dig even [---] those things are called traditional hand dug wells [*Photo 4*]… So, they can dig to up to ten meters… until it reaches the ground level. So they use that water for domestic use [*Photo 5*] and for livestock. Yeah’ (Community Elder, Male, Respondent 4)

**Quote 15:** ‘… [*in Turkana*] … translation… also there are, there are areas [---] designated areas, maybe along a certain seasonal river, whereby during the dry season, they go and [---] dig. They dig a big hole. That maybe even [---] ten people can go inside’ (Community Elder, Male, Respondent 7)

Alternatively, the water sources managed by the government are in the form of boreholes and were of better quality compared to the above-mentioned shallow wells mostly used by pastoralists. A fact understood by the people:

**Quote 16:** ‘…and… if there is drought, that would mean even the quality of water that these people are taking is compromised…’ (Political Leader, Male, Respondent 5)

As described by the participant, when there is drought, the quality of water for pastoralists is compromised. On a different note, in the same community, people attribute the overall morbidities to fate and God:

**Quote 17:** ‘…it is only God taking charge. It is only God that takes charge to the diseases around there…’ (Religious Leader, Male, Respondent 1)

## Modern strategies versus indigenous adaptation

Stemming from the water access and quality, referring to the existing modern systems, for example, water pans, the participants mentioned those created by the non-governmental organisations (NGOs) and maintained by the government, as one of their main sources of water. People are using these for their needs and also for their animals. They also identified some issues with their reliability and maintenance. People feel dependent on the government for the maintenance, which may not happen very adequately. In our discussion with opinion leaders, a participant mentioned:

**Quote 18:** ‘… there are water pan NGOs they came and dig […] they dig when there is rain it collects that water and keep it there… so, when there is drought, that’s when we go and the animals use that water… and even people use that water’ (Opinion Leader, Male, Respondent 1)

In the same discussion, a participant pointed out:

**Quote 19:** ‘[*O*]kay, these water pans… these water… these structures that I am talking about …ahh… the water pans, when these people are using, the animals are drinking from it, the community taking water taking from the same… the wild animals using it, in the process you will find the walls of these pans get eroded inside and it becomes again an issue to de-silt…’ (Opinion Leader, Male, Respondent 7)

As delineated by the participants, the water pans they rely on are the same ones used by their livestock and wild animals, which results in the walls being eroded. Additionally, the participants, in our discussion with political leaders, describing these water pans as ‘unreliable’, stated:

**Quote 20:** ‘… it is again the government to come in to do the de-siltation [*Sic*] or else there is no pan there… so it is not reliable as, as my brother has said… it is not reliable. But with maintenance, regular maintenance, yes it can help… [Another Male participant added]: … which have to be 5 months, 6 months…, five… three… and so on…’ (Political Leaders, Males, Respondents 2 and 4)

This water is then brought home for filtering and treatment using the IK-based practices. Pastoralists rely on ERUT (*Maerua decumbens*) or ABUKUT (*Sansevieria robusta*) to purify their drinking water (Makishima 2005; Watkins 2010). They uproot, smash and stir the dirty water with it until the sediments settle down and then they collect the upper water for domestic use. Demonstrating this traditional method, in our discussion with religious leaders, a participant stated:

**Quote 21:** ‘… and this water [---] they can also possibly use herbs like ERUT [Maerua decumbens] or ABUKUT [Sansevieria robusta] …’ (Religious Leader, Male, Respondent 3)

## Shift from transgenerational livelihoods to survival tactics

Concerning livelihoods, during the study, the participants portrayed pastoralism as the main economic activity for generating income. As elaborated by a participant during our discussion with political leaders:

**Quote 22:** ‘…these people depend on [---] livestock produce, for example milk. Now, when the drought affects the [---] grass and everything and reduce the livestock, then we cannot even have milk which they sell in order to get something, in order to substantiate also with other commodities buying… when we don’t have the milk, definitely, the economy of these people, they cannot be able to purchase anything…’ (Political Leader, Male, Respondent 1)

Because of the deleterious effects of droughts on their livestock, it has also forced them to concentrate their farming activities only on the river sides. But this shifting of focus towards farming comes with certain challenges, as described in this quote:

**Quote 23:** ‘… like these, these pastoralists during this drought, when the drought finishes the animals, now that it becomes a very big problem to them because they are not used to [---] farming, they don’t know how… how to start, when they are given a land to go until… for planting… do preparation for planting… they don’t know…’ (Political Leader, Male, Respondent 7)

As they depend on rain-fed farming, the participants illustrated that they no longer produce various crops such as sorghum, tomatoes, maize and vegetables as before:

**Quote 24:** ‘… the agriculture produce has gone down. So, [---] drought has really affected our… our economy in a negative way’ (Political Leader, Male, Respondent 4)

So because of drought, their agricultural produce has decreased, which negatively affected their economy.

Beyond livestock and farming, another occupation was described during our discussion with community elders:

**Quote 25:** ‘… [*in Turkana*] … translation … it is only those who can burn charcoals [---] and may be who can sell firewood [---] yeah… who can sell firewood, those are the only ones who can survive [---] then the rest they starve. Yeah.’ (Community Elder, Female, Respondent 3)

**PHOTO 6:**
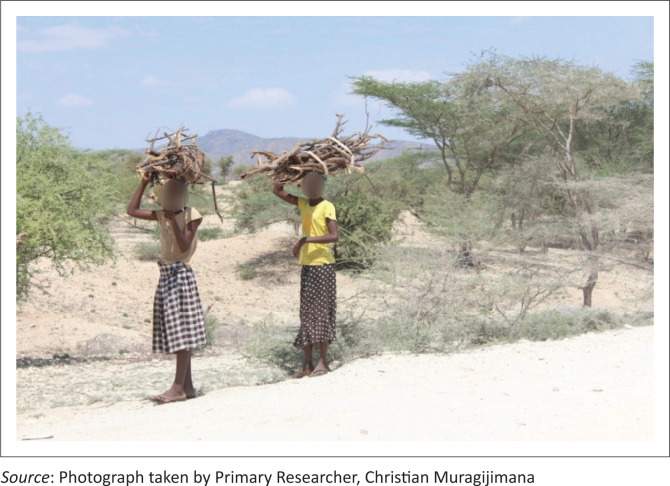
Girls from collecting firewood.

**PHOTO 7:**
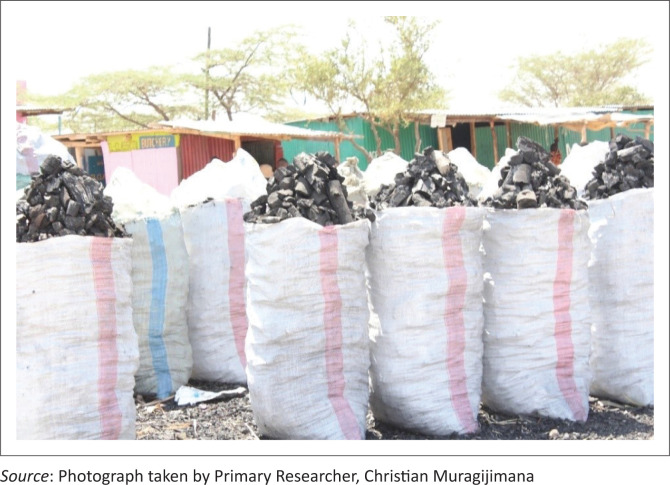
Burned charcoals ready for sale.

As reckoned by the participant, aside from collecting and selling firewood they also burn charcoals to survive (Photo 6 and Photo 7).

Illustrating the reason behind these newly adopted strategies to survive, during our discussion with community elders, participants stated:

**Quote 26:** ‘[*O*]kay, let me also add something…, I think the economic activity of this place is farming. The main economic activity is farming. And …ahh, Pastoralism. They are pastoralists. So, the… the… they, they have, they have subdivided the team to two. There are those who are farming, there are those who are [---] railing animals’ (Community Elder, Male, Respondent 2)

**Quote 27:** ‘[*T*]hey [---] jump to burning charcoals because the farm cannot make…, you cannot, you cannot plant anything. So that is what, that is what makes them go, go to burn charcoal, and also carry firewood. And they don’t do it for business, they don’t do it for business, they are just doing it to sustain, for sustainability, to sustain back their families. Just to sustain the people [---] there. They are not doing it for business’ (Community Elder, Male, Respondent 6)

**Quote 28:** ‘… [*b*]ecause they even, before they didn’t, they were not doing it. It is now drought that has forced them [---] to go that way. It is just drought. … So, it is just because of this drought that has eaten them for the last three years. But [---] long ago, [*n*]o. [*T*]hey were not doing it [*burning charcoals and collecting firewood*] …’ (Community Elder, Male, Respondent 6)

As illustrated, the reason behind the newly adopted strategies to make their end meets and more precisely burning charcoals and collecting firewood is linked to inadequate rainfall as a result of drought, which has negatively impacted faming and pastoralism as major economic activities in Lopur.

## Discussion

This study explored the effects of climate change-induced burden on health and livelihoods. Contrary to Kagunyu et al. (2016), Rukema et al. (2019) and Mashoko et al. (2020) studies that focused on IK as a separate entity, this study investigated IK and related practices through the lens of the required acceptability, suitability and adjustments necessity for sustainable modern DRR strategies in Lopur. Lopur, as the selected study setting, presented the perfect opportunity to learn from on-ground realities related to the current emergency of climate change and drought-driven health and livelihoods effects in particular on the ASALs.

Contrasting with the reviewed literature such as Fatehpanah et al. (2020) and Rankoana (2022), this study classified respondents based on their role and influence in the community. This grouping helped to bring forward some related similarities and dissimilarities. For the similarities and contrary to the examined literature (Iloka 2016; Liberati, Bahta & Jordaan 2018), all the groups, young and old, demonstrated strong beliefs in their IK-based adaptation (quotes 5, 6, 7, 8, 9, 10). Regarding the dissimilarities (quotes 1, 3, 11, 12, 21), the religious group seemed to be more knowledgeable of the modern strategies than political leaders, opinion leaders and community elders. Contrary to Masinde (2015) who underlined religious beliefs to be a limiting factor in the integration necessity of IK in weather forecasts, during this study, the religious group perceived their IK-based practices as critical to adapt to droughts (quotes 10, 21). On the other hand, the community elders were regarded with high esteem by all groups vis-à-vis IK-based information generating and sharing, predictions and preparedness for droughts (quote 5 and its supporting statement).

Further on drought predictions and preparedness information, the participants underpinned their beliefs and reliance on cultural-driven practices compared to scientific methods (compare quotes 1, 2, 3 and 4, 5, 6, 7, 8, 9, 10). As per the study, the key argument reflects around not the disapproval of the modern information systems per se, but more or less limited knowledge of their underlying components that impede the related adoptability (compared quotes 1, 2, 3 and 4, 5, 6, 7, 8, 9, 10). Further, and in accordance with the study by Mutu (2017) and more precisely the illustrated adjustment necessity of modern information dissemination in Turkana, this study underlined that the scientific knowledge was perceived to be superficial and less familiar to the Lopur community (quotes 1, 2, 3). The related modern strategies were illustrated to be a separate entity from the community (outsiders), an aspect against the socio-cultural cohesion that (arguably) would interfere with not only the information but also its meaning and the intended impact (compared quotes 1, 2, 3 and 4, 5, 6, 7, 8, 9, 10).

On the flipside, the participants seemed to resonate much better with their culture and their IK-based system as a source of information to predict and prepare for drought seasons, foretell related diseases, insecurity and external interventions among other benefits (quotes 4, 5, 6, 7, 8, 9, 10). The related basic reasons were twofold:

- as their traditions, they believe that the cultural processes in terms of transmission of information were clear, organised, relevant and relied on by their forefathers (quotes 4, 5, 6, 7, 8, 9, 10); and
- the related usability in terms of system, strategy and preparedness easiness was perceived to be more impactful than modern methods impact (compared quotes 1, 2, 3 and 4, 5, 6, 7, 8, 9, 10).

However, regardless of their reliance on IK and related systems, the respondents did not portray any resistance to modern strategies such as seeking modern healthcare services (quote 12). The portrayed limitations had little to do with beliefs but more about unavailability, inaccessibility and unaffordability of healthcare services as the emphasised limitations to cope with climate change-induced health effects such as waterborne diseases (quote 12).

The underlined waterborne diseases were closely linked to environmentally based water quantity and quality issues (quotes 13, 18, 19, 20). The available sources were perceived as inadequate and unreliable. Water pans, in particular, were perceived by the people to be problematic in terms of quality issues (quotes 18, 19, 20). The reason behind is twofold:

- the water in these pans is stagnant and not protected against external contaminants (quotes 18, 19, 20). The latter alters these intended solutions into the breeding sites of waterborne pathogens; and
- the cultural lifestyle of these communities is considerably nomadic, which makes them move around with animals looking for water for domestic use and their livestock, as well as pasture (referring to the mentioned nomadic lifestyle of the Lopur community).

This led to exacerbated altered patterns and cross-contamination of various diseases between humans, humans to domestic as well as wild animals and vice versa (quotes 18, 19, 20).

In line with the suggestions by Mutu’s (2017) study for resilience prerequisite intersecting climate change vulnerability and nomadic lifestyle of the Turkana communities through the lens of modern interventions, this study pointed out:

- limited local capacity and coordination to take care of modern interventions; and
- sustainability issues and concerns that burden the already existing drought-induced hardships on the Lopur community health and wellbeing.

Contrary to Fatehpanah et al.’s (2020) study that mentioned boiling and use of salt as (arguably) IK based, this study brought to light local IK-based herbs, such as ABUKUT (*Sansevieria robusta*) or ERUT (*Maerua decumbens*) (Makishima 2005; Watkins 2010), of which the roots were relied on to cope with the aforementioned water quality issues (quote 21). On that same note, reflecting on food insecurity, the respondents underpinned reliance on wild fruits, such as ELAMACH (*Balanites pedicullaris*) and EDOME (*Cordia sinensis*) (Watkins 2010), as their IK-based adaptation strategy for survival (quote 10).

To the best of our knowledge, there is (not yet to) limited in-depth evidence for the impact of IK-based alternatives for water quality and treatment such as the use of ABUKUT (*Sansevieria robusta*) and ERUT (*Maerua decumbens*) (Makishima 2005; Watkins 2010). Moreover, there is a need for more research to not only investigate the availability and reliance trends (over time) of drought-friendly bush-grown foods but also their nutritious value and the existing possibilities to maximise their harvest, storage and distribution. This study argues that the latter could probably be critical in building resilience and strengthening local-based capabilities to cope with the effects of recurring droughts amid the growing climate emergency. Further, and in consonance with Rankoana’s (2022) views, the latter could then be sustained by the capacity building based on the unique IK and farming skills to support and improve the related escalation.

However, and contrary to the study by Apraku, Akpan and Moyo (2018) in the South African context that emphasised on the role of IK in maintaining agricultural productivity amid changing climate, and Rankoana’s (2022) views that farming as a new concept cannot yield concrete results if not intertwined with local realities, this study underlined that droughts have forced the Lopur community to adopt farming practices that, as a new concept, they struggle on how to apply this transition amid the rising climate change (quote 23). This disconnect exacerbates their vulnerability and reliance on the governmental support perceived as inopportune, besides the rising adopted survival strategies (and arguably) environmental degrading tactics, such as cutting trees to burn charcoals and sell firewood in order to cope with droughts (quotes 26, 27, 28). The latter emphasises the necessity to integrate IK contextual realities and variabilities to ensure a more impactful IK-based adaptation to climate change.

According to Leal Filho et al. (2022), IK is not only critical for successful and cost-effective modern interventions in resource-constraint settings, but also reinforces the sense of ownership and the related sustainability. In the same vein, the contextualised insights from Lopur give a glimpse into the necessity to incorporate IK as another area of expertise to address climate burden on health, through information dissemination and use for more robust and fostered warning systems and preparedness for drought in the poor resource constraints such as ASALs.

**FIGURE 3:**
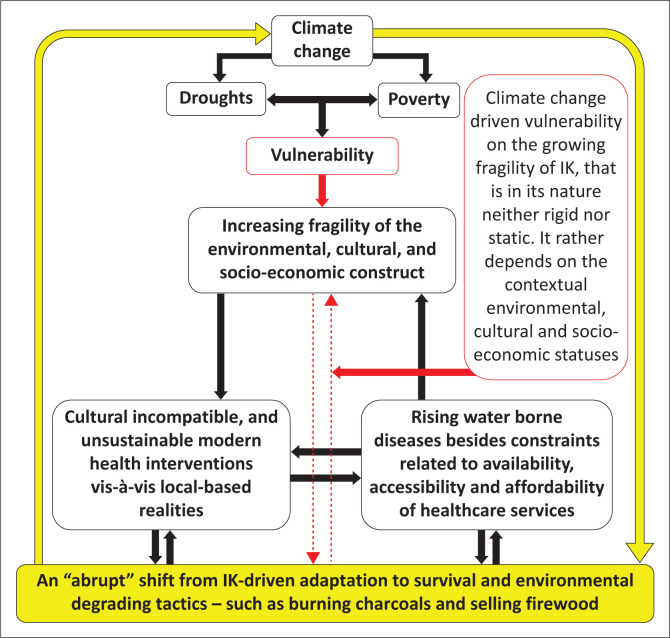
Climate change-driven vulnerability.

This study recommends that future researchers further explore the impact of external support (and its related possibility for counterproductive effect) on the prerequisite local resilience amid the current growing climate emergency. Moreover, and in reference to Figure 3, the perceived climate change-driven vulnerability to investigate drought-induced shift from core economic activities (farming and pastoralism) to the adopted environmental degrading survival tactics (such as burning charcoals) and the impact of IK-based adaptation on drought-induced water and food insecurity in the ASALs’s vulnerable drought-prone rural communities in low-resource settings.

Nevertheless, the intent is not to portray the IK as the ‘forgotten right stack of knowledge’ complete and enough to provide the adaptation capabilities to climate change on its own. Instead, and concurring with arguments by Apraku et al. (2018), Jessen et al. (2022), Zvobgo et al. (2022) and Kom et al. (2023), this study attempts to underline IK necessity and prerequisites components to foster and make our understanding more holistic.

In consonance with Mutu (2017) who underlined poor modern information dissemination that impedes the related uptake by the nomadic pastoral communities in Turkana, this study underlined the key message for health intervention providers to ensure cultural integration and incorporation of indigenous systems to improve the information dissemination, accessibility and acceptability through the community. However, it remains critical to underline that there is a need for more research to ensure the reliability of these concrete IK-based strategies to sustainably alleviate health burden and ecological threats on the most vulnerable, remote agropastoral communities in the ASALs who bear an inequitable burden of climate change.

This study had some limitations:

- The ideal plan during the study was to have half males and half females as participants. The study recognises that this could probably have provided more insights on the determinants of the shared experiences. However, this goal was not achieved. Presumably intended as a possible traditional hiccup that dictates males and females’ interaction in this specific cultural setting, the later had a limited effect in the completion of this study.
- Prior to data collection, the team was informed that the first group discussion with community elders will be exclusively in Turkana language. The latter was partially against the original plan. However, as the PR already pre-planned to be an outsider during this specific FGD, the team ensured on-field clarification of the shared insights by making sure that the conversation was not only between the translator and the participants but also between the PR and the translator.

On the other hand, this study portrayed some notable strengths:

- Lopur, as our study setting and a community transitioning from pure nomadic lifestyle to a settled and semi-settled lifestyle, presented the perfect opportunity to learn from on-ground realities and fitted perfectly with the intent of this study.
- The selection of the translator reflected personal knowledge by the PR. The translator took the lead in the selection of the local mobiliser. Fluent in Turkana, Swahili and English, both the translator and local mobiliser were born and raised in Turkana with similar cultural background as the study participants that created the role of an insider and helped to build confidence with the participants.
- The PR allocated 7 days to ensure the unanimity in research activities and responsibilities and made sure that the translator and the local mobiliser adopt to not only the purpose of this study but also its required smooth feasibility and early planning to minimise unpredictable issues while at the field.

The latter helped for effective and appropriate preparations, comfortability of the participants and influenced the shared detailed, extensive opinions and experiences related to the questions of interest by the participants who presented themselves at time at the designated location (within proximity of each participant) that was proven by active participation.

Furthermore, none of the participants dropped out of the study and none left (for any other personal reasons) regardless of the indisputability aspect to voluntarily participate clearly described prior to conducting this study and followed by provided verbal consent by the participants captured on records:

- The PR conducted all the FGDs that ensured consistency in the adopted styles and techniques and data analysis, in close collaboration with the translator, double-checked by the core research team and wrote the report that increased validity and eliminated possible biases during the study.

## Recommendations for research, practice and policy

This study cast lights on the necessity of cultural significance of health as a bridging element for the seeming disconnect between climate change-induced health burden and livelihood threats, unsustainable modern interventions and the rising concern associated with the adopted survival (arguably environmental degrading) tactics. Indigenous knowledge incorporation prerequisite into modern DRR strategies might be argued to be crucial to ensure not only their adoptability but also sustainability.

The study pinpointed the necessity of IK system in the flow or dissemination of information. Further, there seems to be a thin line between information dissemination and adoptability by the community. The study indicated that there is a need to incorporate local IK and systems to foster and improve accessibility and acceptability of information related to climate change adaptation. Thus, IK incorporation, as another area of expertise, remains critical to inform inclusive and sustainable preparedness and response strategies to climate change and related disasters such as droughts.

On policy, practice and research and based on the acquired insights, the outlined recommendations can be achieved through three strategies:

1. Involvement of the religious leaders, kraal or community elders into decision making, planning and implementation of interventions for climate change adaptation. Further, community engagement alongside reinforced local capacities as a cultural-based bi-directional approach that involves diversification of economy remains critical to create locally adoptable survival alternatives. The latter would help to reinforce and align modern systems to the local tradition-based lifestyles, beliefs and cultural-based capacities to sustain provided modern interventions.
2. The study portrayed the need for policy and practice to address the mostly unknown (or ignored) cultural risks and gaps that tend to reverse the intended impact of modern solutions into deeper and ravaging health concerns.
3. Research relevant, in accordance with Malapane et al. (2022) the study portrayed the existing room for scientific exploration of the already existing IK-based solutions for food and water insecurity, beyond (the common) contextual perspectives and tailor them towards not only addressing the rising health and livelihoods fragility as a result of climate change but also building a more drought-resilient community amid the teeth of climate change in the ASALs.

## Conclusion

The contextualised insights from Lopur revealed the necessity to incorporate cultural significance of health as part of the modern DRR strategies. The findings delineated the indispensability to incorporate local-based cultural practices and IK alongside the ‘required modern adjustments’ to reflect the on-ground realities and reinforce information dissemination. Indigenous knowledge -driven information dissemination is critical for related accessibility and acceptability to not only address the ravaging effects of droughts on health and livelihoods but also the growing social vulnerability, as a result of the disconnect between modern interventions, IK and the newly adopted (unsustainable and environmentally degrading) local-based coping tactics.

Through the lens of the putative policy and research implications, this study lays the need for a cultural-based bi-directional model to sustainably address water and food insecurity, through adoption and implementation of locally adoptable survival alternatives, community involvement and engagement. These findings cast light on the key element for the indispensability to strengthen the emphasised interdependence nature of both expertise (indigenous knowledge-based adaptation and modern scientific-based adaptation strategies).

It provides the basis as well as the need for more scientific exploration of IK as another area of expertise. The intent is not to portray IK as the ‘panacea’ for all challenges to climate change adaptation, but rather to ensure mutual complementation with the current modern strategies, systems and tools, to improve health, wellbeing and reinforce resilience of the most vulnerable communities, experiencing inequitable climate change calamities in the ASALs.
